# Bis­(*N*,*N*′-diphenyl­thio­urea)iodido­copper(I) monohydrate

**DOI:** 10.1107/S1600536808007861

**Published:** 2008-05-03

**Authors:** Li Jia, Lingqian Kong, Dacheng Li

**Affiliations:** a Liaocheng Vocational and Technical College, Liaocheng, Shandong 252000, People’s Republic of China; b Dongchang College of Liaocheng University, Liaocheng, Shandong 252000, People’s Republic of China; c School of Chemistry and Chemical Engineering, Liaocheng University, Shandong 252059, People’s Republic of China

## Abstract

In the title compound, [CuI(C_13_H_12_N_2_S)_2_]·H_2_O, each Cu(I) ion is coordinated by two S atoms [Cu—S 2.2282 (16), 2.2377 (15) Å] from two *N*,*N*′-diphenyl­thio­urea ligands and one iodide ion [Cu—I 2.5170 (11) Å] in a trigonal planar geometry. The uncoordinated water mol­ecules are involved in N—H⋯O hydrogen-bonding [N⋯O 2.947 (5), 3.055 (5) Å], which link the mol­ecules into chains extended in the [101] direction. These chains are further paired by weak inter­molecular O—H⋯S hydrogen bonds [O⋯S 3.490 (4) Å].

## Related literature

For geometrical parameters in related crystal structures, see: Lobana *et al.* (2006 ▶).
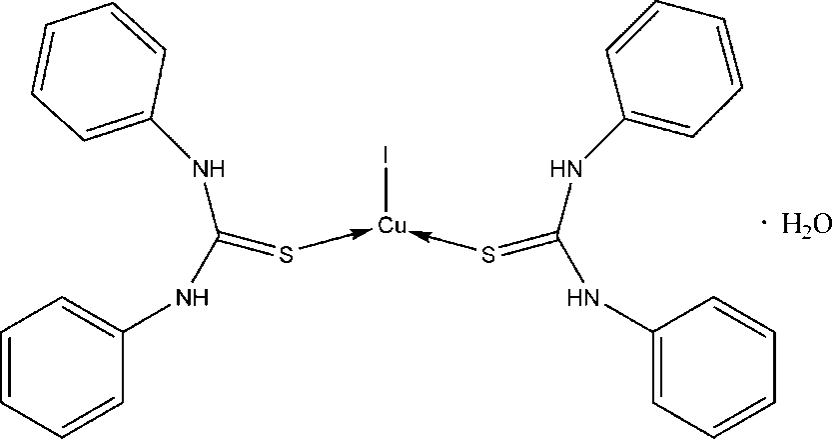


## Experimental

### 

#### Crystal data

[CuI(C_13_H_12_N_2_S)_2_]·H_2_O
*M* *_r_* = 665.07
Triclinic, 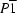

*a* = 9.700 (4) Å
*b* = 12.490 (5) Å
*c* = 12.935 (5) Å
α = 91.489 (5)°
β = 108.110 (5)°
γ = 110.950 (5)°
*V* = 1374.4 (9) Å^3^
*Z* = 2
Mo *K*α radiation
μ = 2.10 mm^−1^
*T* = 298 (2) K
0.28 × 0.19 × 0.18 mm

#### Data collection

Bruker SMART CCD area-detector diffractometer
Absorption correction: multi-scan (*SADABS*; Sheldrick, 1996 ▶) *T*_min_ = 0.592, *T*_max_ = 0.704
7290 measured reflections
4804 independent reflections
2999 reflections with *I* > 2σ(*I*)
*R*_int_ = 0.031

#### Refinement

*R*[*F* ^2^ > 2σ(*F* ^2^)] = 0.041
*wR*(*F* ^2^) = 0.085
*S* = 0.87
4804 reflections
316 parameters
H-atom parameters constrained
Δρ_max_ = 0.57 e Å^−3^
Δρ_min_ = −0.45 e Å^−3^

### 

Data collection: *SMART* (Siemens, 1996 ▶); cell refinement: *SAINT* (Siemens, 1996 ▶); data reduction: *SAINT*; program(s) used to solve structure: *SHELXS97* (Sheldrick, 2008 ▶); program(s) used to refine structure: *SHELXL97* (Sheldrick, 2008 ▶); molecular graphics: *SHELXTL* (Sheldrick, 2008 ▶); software used to prepare material for publication: *SHELXTL*.

## Supplementary Material

Crystal structure: contains datablocks I, global. DOI: 10.1107/S1600536808007861/cv2394sup1.cif

Structure factors: contains datablocks I. DOI: 10.1107/S1600536808007861/cv2394Isup2.hkl

Additional supplementary materials:  crystallographic information; 3D view; checkCIF report

## Figures and Tables

**Table 1:** Hydrogen-bond geometry (Å, °)

*D*—H⋯*A*	*D*—H	H⋯*A*	*D*⋯*A*	*D*—H⋯*A*
N1—H1⋯I1	0.86	2.87	3.706 (4)	166
N2—H2⋯O1^i^	0.86	2.14	2.947 (5)	156
N3—H3⋯I1	0.86	2.82	3.666 (4)	168
N4—H4⋯O1^ii^	0.86	2.38	3.055 (5)	136
O1—H1*B*⋯S2^iii^	0.85	2.64	3.490 (4)	179

Symmetry codes: (i) 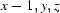; (ii) 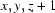; (iii) 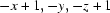.

**Fig. 1.**
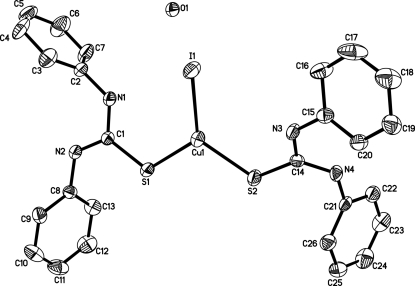
The molecular structure of (I), showing the atomic numbering and 40% probability displacement ellipsoids. H atoms omitted for clarity.

**Fig. 2.**
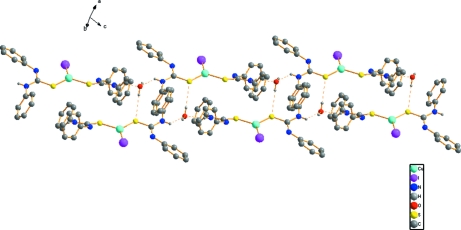
A portion of the crystal packing showing the paired hydrogen-bonded (dashed lines) chains. H atoms not involved in hydrogen-bonding are omitted for clarity.

## supplementary crystallographic information

### Comment

In this paper, we report the synthesis and the crystal structure of the title compound (I).

In (I) (Fig. 1), the Cu(I) ion is in a trigonal coordination environment formed by to two S atoms of two monodentate diphenylthiourea ligands and one iodine ion. The Cu-S [2.2282 (16), 2.2377 (15) Å] and Cu-I [2.5170 (11) Å] bond lengths agree well with those reported for the related compounds (Lobana *et al.*, 2006).

The crystalline water molecules are involved in N—H···O hydrogen-bonding (Table 1), which link the molecules into chains extended in direction [101]. These chains are further paired (Fig. 2) by the weak intermolecular O—H···S hydrogen bonds (Table 1).

### Experimental

CuI (0.19 g 1 mmol) and diphenylthiourea (0.46 g 2 mmol) in 10 ml acetonitrile,refluxed for 24 h, then a colourless solution formed. After filtration, the solution was allowed to evaporate slowly. Crystals suitable for X-ray diffraction were obtained after several days.

### Refinement

All H atoms were placed in calculated positions (O-H 0.85 Å, N-H 0.86 Å, C-H 0.93 Å), and treated as riding on their parent atoms, with  *U*_iso_(H) = 1.2*U*_eq_ of the parent atoms.

### Crystal data

**Table:**

[CuI(C_13_H_12_N_2_S)_2_]·H_2_O	*Z* = 2
*M**_r_* = 665.07	*F*_000_ = 664
Triclinic, *P*1	*D*_x_ = 1.607 Mg m^−^^3^
*a* = 9.700 (4) Å	Mo *K*α radiation λ = 0.71073 Å
*b* = 12.490 (5) Å	Cell parameters from 1949 reflections
*c* = 12.935 (5) Å	θ = 2.4–22.1º
α = 91.489 (5)º	µ = 2.10 mm^−^^1^
β = 108.110 (5)º	*T* = 298 (2) K
γ = 110.950 (5)º	Block, colourless
*V* = 1374.4 (9) Å^3^	0.28 × 0.19 × 0.18 mm

### Data collection

**Table:**

Bruker SMART CCD area-detector diffractometer	4804 independent reflections
Radiation source: fine-focus sealed tube	2999 reflections with *I* > 2σ(*I*)
Monochromator: graphite	*R*_int_ = 0.031
*T* = 298(2) K	θ_max_ = 25.0º
phi and ω scans	θ_min_ = 1.7º
Absorption correction: multi-scan(SADABS; Sheldrick, 1996)	*h* = −11→11
*T*_min_ = 0.592, *T*_max_ = 0.704	*k* = −14→14
7290 measured reflections	*l* = −15→9

### Refinement

**Table:**

Refinement on *F*^2^	Secondary atom site location: difference Fourier map
Least-squares matrix: full	Hydrogen site location: inferred from neighbouring sites
*R*[*F*^2^ > 2σ(*F*^2^)] = 0.041	H-atom parameters constrained
*wR*(*F*^2^) = 0.085	*w* = 1/[σ^2^(*F*_o_^2^) + (0.0329*P*)^2^] where *P* = (*F*_o_^2^ + 2*F*_c_^2^)/3
*S* = 0.87	(Δ/σ)_max_ < 0.001
4804 reflections	Δρ_max_ = 0.58 e Å^−^^3^
316 parameters	Δρ_min_ = −0.45 e Å^−^^3^
Primary atom site location: structure-invariant direct methods	Extinction correction: none

### Special details

**Table:**

Geometry. All e.s.d.'s (except the e.s.d. in the dihedral angle between two l.s. planes) are estimated using the full covariance matrix. The cell e.s.d.'s are taken into account individually in the estimation of e.s.d.'s in distances, angles and torsion angles; correlations between e.s.d.'s in cell parameters are only used when they are defined by crystal symmetry. An approximate (isotropic) treatment of cell e.s.d.'s is used for estimating e.s.d.'s involving l.s. planes.
Refinement. Refinement of *F*^2^ against ALL reflections. The weighted *R*-factor *wR* and goodness of fit *S* are based on *F*^2^, conventional *R*-factors *R* are based on *F*, with *F* set to zero for negative *F*^2^. The threshold expression of *F*^2^ > σ(*F*^2^) is used only for calculating *R*-factors(gt) *etc*. and is not relevant to the choice of reflections for refinement. *R*-factors based on *F*^2^ are statistically about twice as large as those based on *F*, and *R*- factors based on ALL data will be even larger.

### Fractional atomic coordinates and isotropic or equivalent isotropic displacement parameters (Å^2^)

**Table:**

	*x*	*y*	*z*	*U*_iso_*/*U*_eq_	
Cu1	0.05943 (7)	0.11385 (5)	0.77393 (5)	0.04630 (19)	
I1	0.17712 (4)	0.33136 (3)	0.82406 (3)	0.05399 (14)	
N1	−0.0381 (4)	0.2061 (3)	0.5295 (3)	0.0443 (11)	
H1	0.0273	0.2349	0.5952	0.053*	
N2	−0.2538 (4)	0.0540 (3)	0.4163 (3)	0.0436 (11)	
H2	−0.2571	0.0987	0.3669	0.052*	
N3	0.2680 (4)	0.1602 (3)	1.0425 (3)	0.0405 (10)	
H3	0.2322	0.1966	0.9921	0.049*	
N4	0.3157 (4)	−0.0053 (3)	1.0771 (3)	0.0406 (10)	
H4	0.3845	0.0350	1.1385	0.049*	
O1	0.6610 (4)	0.1414 (3)	0.2058 (3)	0.0619 (11)	
H1A	0.6840	0.2105	0.1920	0.074*	
H1B	0.7159	0.1107	0.1858	0.074*	
S1	−0.12503 (15)	0.01325 (10)	0.61534 (11)	0.0466 (4)	
S2	0.11547 (15)	−0.01538 (10)	0.87828 (11)	0.0474 (4)	
C1	−0.1409 (5)	0.0964 (4)	0.5129 (4)	0.0347 (12)	
C2	−0.0288 (6)	0.2790 (4)	0.4464 (4)	0.0423 (13)	
C3	−0.1082 (7)	0.3512 (4)	0.4296 (5)	0.0613 (16)	
H3A	−0.1706	0.3526	0.4711	0.074*	
C4	−0.0948 (8)	0.4228 (5)	0.3497 (5)	0.073 (2)	
H4A	−0.1478	0.4729	0.3384	0.088*	
C5	−0.0044 (7)	0.4203 (5)	0.2876 (5)	0.0701 (19)	
H5	0.0043	0.4687	0.2345	0.084*	
C6	0.0738 (8)	0.3459 (6)	0.3040 (6)	0.085 (2)	
H6	0.1342	0.3424	0.2615	0.102*	
C7	0.0606 (6)	0.2770 (5)	0.3844 (5)	0.0645 (17)	
H7	0.1145	0.2275	0.3966	0.077*	
C8	−0.3704 (5)	−0.0610 (4)	0.3884 (4)	0.0379 (12)	
C9	−0.5254 (6)	−0.0777 (4)	0.3680 (4)	0.0470 (14)	
H9	−0.5529	−0.0142	0.3729	0.056*	
C10	−0.6383 (6)	−0.1869 (5)	0.3406 (5)	0.0624 (17)	
H10	−0.7425	−0.1977	0.3278	0.075*	
C11	−0.5994 (7)	−0.2808 (5)	0.3318 (5)	0.0687 (18)	
H11	−0.6772	−0.3553	0.3124	0.082*	
C12	−0.4450 (8)	−0.2653 (5)	0.3517 (5)	0.0665 (18)	
H12	−0.4180	−0.3290	0.3465	0.080*	
C13	−0.3309 (6)	−0.1546 (4)	0.3791 (5)	0.0512 (14)	
H13	−0.2267	−0.1435	0.3914	0.061*	
C14	0.2398 (5)	0.0499 (4)	1.0080 (4)	0.0342 (12)	
C15	0.3482 (5)	0.2268 (4)	1.1501 (5)	0.0412 (13)	
C16	0.4300 (7)	0.3453 (4)	1.1573 (5)	0.0581 (16)	
H16	0.4354	0.3778	1.0941	0.070*	
C17	0.5030 (8)	0.4140 (5)	1.2591 (6)	0.081 (2)	
H17	0.5568	0.4935	1.2646	0.097*	
C18	0.4967 (7)	0.3661 (5)	1.3517 (6)	0.0724 (19)	
H18	0.5469	0.4131	1.4201	0.087*	
C19	0.4175 (6)	0.2495 (5)	1.3452 (5)	0.0553 (15)	
H19	0.4151	0.2173	1.4090	0.066*	
C20	0.3410 (6)	0.1801 (4)	1.2436 (4)	0.0444 (13)	
H20	0.2844	0.1012	1.2388	0.053*	
C21	0.2935 (6)	−0.1242 (4)	1.0585 (4)	0.0371 (12)	
C22	0.4230 (6)	−0.1510 (4)	1.0756 (4)	0.0492 (14)	
H22	0.5229	−0.0927	1.0974	0.059*	
C23	0.4029 (7)	−0.2666 (5)	1.0598 (5)	0.0631 (17)	
H23	0.4903	−0.2856	1.0711	0.076*	
C24	0.2569 (8)	−0.3529 (5)	1.0281 (5)	0.0699 (18)	
H24	0.2448	−0.4300	1.0170	0.084*	
C25	0.1277 (7)	−0.3252 (5)	1.0125 (5)	0.0673 (18)	
H25	0.0279	−0.3838	0.9910	0.081*	
C26	0.1456 (6)	−0.2107 (4)	1.0286 (5)	0.0498 (14)	
H26	0.0583	−0.1920	1.0193	0.060*	

### Atomic displacement parameters (Å^2^)

**Table:**

	*U*^11^	*U*^22^	*U*^33^	*U*^12^	*U*^13^	*U*^23^
Cu1	0.0492 (4)	0.0489 (4)	0.0387 (4)	0.0194 (3)	0.0111 (3)	0.0147 (3)
I1	0.0693 (3)	0.0439 (2)	0.0507 (3)	0.02398 (19)	0.0197 (2)	0.01702 (19)
N1	0.044 (2)	0.041 (2)	0.033 (3)	0.010 (2)	0.001 (2)	0.011 (2)
N2	0.043 (2)	0.040 (2)	0.039 (3)	0.013 (2)	0.005 (2)	0.013 (2)
N3	0.053 (3)	0.037 (2)	0.030 (3)	0.019 (2)	0.008 (2)	0.010 (2)
N4	0.048 (2)	0.035 (2)	0.028 (2)	0.015 (2)	0.001 (2)	0.001 (2)
O1	0.065 (2)	0.056 (2)	0.050 (3)	0.017 (2)	0.008 (2)	0.010 (2)
S1	0.0489 (8)	0.0414 (8)	0.0394 (8)	0.0117 (6)	0.0077 (7)	0.0152 (7)
S2	0.0557 (8)	0.0400 (7)	0.0360 (8)	0.0166 (7)	0.0037 (7)	0.0076 (7)
C1	0.037 (3)	0.035 (3)	0.033 (3)	0.015 (2)	0.012 (3)	0.008 (2)
C2	0.046 (3)	0.037 (3)	0.041 (3)	0.015 (3)	0.011 (3)	0.014 (3)
C3	0.080 (4)	0.057 (4)	0.058 (4)	0.036 (3)	0.028 (4)	0.014 (3)
C4	0.112 (5)	0.057 (4)	0.066 (5)	0.052 (4)	0.025 (4)	0.032 (4)
C5	0.092 (5)	0.050 (4)	0.058 (4)	0.018 (3)	0.021 (4)	0.029 (3)
C6	0.103 (5)	0.104 (5)	0.086 (6)	0.054 (5)	0.062 (5)	0.057 (5)
C7	0.074 (4)	0.077 (4)	0.070 (5)	0.046 (4)	0.039 (4)	0.044 (4)
C8	0.039 (3)	0.041 (3)	0.029 (3)	0.009 (2)	0.012 (3)	0.006 (2)
C9	0.042 (3)	0.056 (3)	0.041 (4)	0.020 (3)	0.011 (3)	0.007 (3)
C10	0.041 (3)	0.071 (4)	0.062 (4)	0.007 (3)	0.020 (3)	−0.006 (4)
C11	0.064 (4)	0.057 (4)	0.059 (4)	−0.005 (3)	0.020 (4)	−0.007 (3)
C12	0.086 (5)	0.044 (4)	0.070 (5)	0.026 (4)	0.028 (4)	−0.003 (3)
C13	0.052 (3)	0.052 (4)	0.052 (4)	0.020 (3)	0.022 (3)	0.001 (3)
C14	0.034 (3)	0.032 (3)	0.032 (3)	0.007 (2)	0.012 (2)	0.004 (2)
C15	0.043 (3)	0.035 (3)	0.047 (4)	0.015 (2)	0.018 (3)	0.006 (3)
C16	0.082 (4)	0.040 (3)	0.055 (4)	0.013 (3)	0.039 (4)	0.009 (3)
C17	0.097 (5)	0.039 (3)	0.090 (6)	−0.008 (3)	0.050 (5)	−0.015 (4)
C18	0.084 (5)	0.057 (4)	0.061 (5)	0.006 (4)	0.031 (4)	−0.017 (4)
C19	0.057 (4)	0.058 (4)	0.043 (4)	0.015 (3)	0.016 (3)	0.000 (3)
C20	0.047 (3)	0.039 (3)	0.045 (4)	0.011 (3)	0.018 (3)	0.006 (3)
C21	0.050 (3)	0.037 (3)	0.026 (3)	0.018 (3)	0.013 (3)	0.012 (2)
C22	0.049 (3)	0.057 (4)	0.045 (4)	0.023 (3)	0.017 (3)	0.014 (3)
C23	0.081 (5)	0.070 (4)	0.065 (5)	0.046 (4)	0.039 (4)	0.022 (4)
C24	0.098 (5)	0.045 (4)	0.069 (5)	0.038 (4)	0.019 (4)	0.007 (3)
C25	0.064 (4)	0.043 (4)	0.077 (5)	0.009 (3)	0.013 (4)	0.017 (3)
C26	0.050 (3)	0.046 (3)	0.058 (4)	0.022 (3)	0.019 (3)	0.016 (3)

### Geometric parameters (Å, °)

**Table:**

Cu1—S1	2.2282 (16)	C9—C10	1.360 (7)
Cu1—S2	2.2377 (15)	C9—H9	0.9300
Cu1—I1	2.5170 (11)	C10—C11	1.367 (7)
N1—C1	1.338 (5)	C10—H10	0.9300
N1—C2	1.431 (6)	C11—C12	1.377 (7)
N1—H1	0.8600	C11—H11	0.9300
N2—C1	1.320 (6)	C12—C13	1.378 (7)
N2—C8	1.425 (5)	C12—H12	0.9300
N2—H2	0.8600	C13—H13	0.9300
N3—C14	1.340 (5)	C15—C20	1.367 (7)
N3—C15	1.427 (6)	C15—C16	1.392 (7)
N3—H3	0.8600	C16—C17	1.378 (8)
N4—C14	1.343 (5)	C16—H16	0.9300
N4—C21	1.424 (5)	C17—C18	1.362 (8)
N4—H4	0.8600	C17—H17	0.9300
O1—H1A	0.8500	C18—C19	1.367 (7)
O1—H1B	0.8500	C18—H18	0.9300
S1—C1	1.708 (5)	C19—C20	1.380 (7)
S2—C14	1.703 (5)	C19—H19	0.9300
C2—C7	1.358 (7)	C20—H20	0.9300
C2—C3	1.363 (6)	C21—C22	1.368 (6)
C3—C4	1.389 (7)	C21—C26	1.379 (6)
C3—H3A	0.9300	C22—C23	1.386 (7)
C4—C5	1.368 (8)	C22—H22	0.9300
C4—H4A	0.9300	C23—C24	1.364 (7)
C5—C6	1.377 (8)	C23—H23	0.9300
C5—H5	0.9300	C24—C25	1.373 (7)
C6—C7	1.375 (7)	C24—H24	0.9300
C6—H6	0.9300	C25—C26	1.379 (7)
C7—H7	0.9300	C25—H25	0.9300
C8—C13	1.367 (6)	C26—H26	0.9300
C8—C9	1.378 (6)		
			
S1—Cu1—S2	106.87 (6)	C10—C11—C12	120.0 (5)
S1—Cu1—I1	125.81 (4)	C10—C11—H11	120.0
S2—Cu1—I1	127.32 (5)	C12—C11—H11	120.0
C1—N1—C2	125.0 (4)	C11—C12—C13	119.5 (5)
C1—N1—H1	117.5	C11—C12—H12	120.2
C2—N1—H1	117.5	C13—C12—H12	120.2
C1—N2—C8	124.3 (4)	C8—C13—C12	120.2 (5)
C1—N2—H2	117.8	C8—C13—H13	119.9
C8—N2—H2	117.8	C12—C13—H13	119.9
C14—N3—C15	130.0 (4)	N3—C14—N4	118.1 (4)
C14—N3—H3	115.0	N3—C14—S2	120.2 (4)
C15—N3—H3	115.0	N4—C14—S2	121.6 (3)
C14—N4—C21	126.3 (4)	C20—C15—C16	119.8 (5)
C14—N4—H4	116.8	C20—C15—N3	122.9 (4)
C21—N4—H4	116.8	C16—C15—N3	117.3 (5)
H1A—O1—H1B	110.0	C17—C16—C15	119.3 (6)
C1—S1—Cu1	112.56 (17)	C17—C16—H16	120.3
C14—S2—Cu1	111.44 (16)	C15—C16—H16	120.3
N2—C1—N1	118.9 (4)	C18—C17—C16	120.2 (5)
N2—C1—S1	120.9 (4)	C18—C17—H17	119.9
N1—C1—S1	120.3 (4)	C16—C17—H17	119.9
C7—C2—C3	119.7 (5)	C17—C18—C19	120.7 (6)
C7—C2—N1	119.9 (4)	C17—C18—H18	119.6
C3—C2—N1	120.4 (5)	C19—C18—H18	119.6
C2—C3—C4	119.3 (6)	C18—C19—C20	119.6 (6)
C2—C3—H3A	120.4	C18—C19—H19	120.2
C4—C3—H3A	120.4	C20—C19—H19	120.2
C5—C4—C3	120.7 (5)	C15—C20—C19	120.3 (5)
C5—C4—H4A	119.7	C15—C20—H20	119.9
C3—C4—H4A	119.7	C19—C20—H20	119.9
C4—C5—C6	119.8 (6)	C22—C21—C26	120.6 (5)
C4—C5—H5	120.1	C22—C21—N4	118.5 (4)
C6—C5—H5	120.1	C26—C21—N4	120.9 (4)
C7—C6—C5	118.7 (6)	C21—C22—C23	119.0 (5)
C7—C6—H6	120.7	C21—C22—H22	120.5
C5—C6—H6	120.7	C23—C22—H22	120.5
C2—C7—C6	121.9 (5)	C24—C23—C22	120.9 (5)
C2—C7—H7	119.0	C24—C23—H23	119.5
C6—C7—H7	119.0	C22—C23—H23	119.5
C13—C8—C9	119.7 (5)	C23—C24—C25	119.7 (5)
C13—C8—N2	120.8 (4)	C23—C24—H24	120.2
C9—C8—N2	119.5 (4)	C25—C24—H24	120.2
C10—C9—C8	120.2 (5)	C24—C25—C26	120.1 (5)
C10—C9—H9	119.9	C24—C25—H25	119.9
C8—C9—H9	119.9	C26—C25—H25	119.9
C9—C10—C11	120.4 (5)	C21—C26—C25	119.6 (5)
C9—C10—H10	119.8	C21—C26—H26	120.2
C11—C10—H10	119.8	C25—C26—H26	120.2

### Hydrogen-bond geometry (Å, °)

**Table:**

*D*—H···*A*	*D*—H	H···*A*	*D*···*A*	*D*—H···*A*
N1—H1···I1	0.86	2.87	3.706 (4)	166
N2—H2···O1^i^	0.86	2.14	2.947 (5)	156
N3—H3···I1	0.86	2.82	3.666 (4)	168
N4—H4···O1^ii^	0.86	2.38	3.055 (5)	136
O1—H1B···S2^iii^	0.85	2.64	3.490 (4)	179

Symmetry codes: (i) *x*−1, *y*, *z*; (ii) *x*, *y*, *z*+1; (iii) −*x*+1, −*y*, −*z*+1.

## References

[bb2] Lobana, T. S., Khanna, S., Butcher, R. J., Hunter, A. D. & Zeller, M. (2006). *Polyhedron*, **25**, 2755–2763.

[bb3] Sheldrick, G. M. (1996). *SADABS* University of Göttingen, Germany.

[bb4] Sheldrick, G. M. (2008). *Acta Cryst.* A**64**, 112–122. 10.1107/S0108767307043930 18156677

[bb1] Siemens (1996). *SMART* and *SAINT* Bruker AXS Inc., Madison, Wisconsin, USA.

